# Deep learning predicts chromosomal instability from histopathology images

**DOI:** 10.1016/j.isci.2021.102394

**Published:** 2021-04-03

**Authors:** Zhuoran Xu, Akanksha Verma, Uska Naveed, Samuel F. Bakhoum, Pegah Khosravi, Olivier Elemento

**Affiliations:** 1 Caryl and Israel Englander Institute for Precision Medicine, Weill Cornell Medicine, New York 10065, USA; 2 Pathology and Laboratory Medicine, Weill Cornell Medicine, New York 10065, USA; 3 Human Oncology and Pathogenesis Program, Memorial Sloan Kettering Cancer Center, New York 10021, USA; 4 Department of Radiation Oncology, Memorial Sloan Kettering Cancer Center, New York 10021, USA; 5 Computational Oncology, Department of Epidemiology and Biostatistics, Memorial Sloan Kettering Cancer Center, New York 10021, USA

**Keywords:** Cell Biology, Automation in Bioinformatics, Neural Networks, Cancer Systems Biology

## Abstract

Chromosomal instability (CIN) is a hallmark of human cancer yet not readily testable for patients with cancer in routine clinical setting. In this study, we sought to explore whether CIN status can be predicted using ubiquitously available hematoxylin and eosin histology through a deep learning-based model. When applied to a cohort of 1,010 patients with breast cancer (Training set: n = 858, Test set: n = 152) from The Cancer Genome Atlas where 485 patients have high CIN status, our model accurately classified CIN status, achieving an area under the curve of 0.822 with 81.2% sensitivity and 68.7% specificity in the test set. Patch-level predictions of CIN status suggested intra-tumor heterogeneity within slides. Moreover, presence of patches with high predicted CIN score within an entire slide was more predictive of clinical outcome than the average CIN score of the slide, thus underscoring the clinical importance of intra-tumor heterogeneity.

## Highlights

• Deep learning model accurately predicts CIN from histopathology slides
• There is evidence for CIN intra-tumor heterogeneity with prognostic value
• CIN is associated with profound transcriptional changes including mitotic pathways
• Results pave the way for using CIN as a prognosis biomarker

## Graphical abstract

**Figure:**
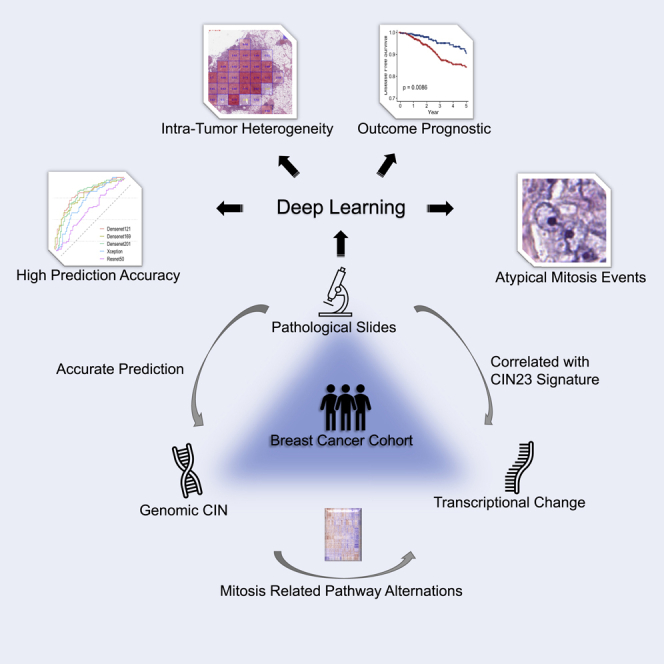

## Introduction

Chromosomal instability (CIN) refers to ongoing chromosome segregation errors throughout consecutive cell divisions that can potentially result in extensive numerical and structural chromosomal aberrations (Lengauer et al. 1998). CIN, as one of the hallmarks of human cancer, has been recognized as a central driver of cancer evolution owning to its multipronged effects that facilitate processes such as metastasis, immune evasion, and therapeutic resistance (Bakhoum and Cantley 2018; Bakhom 2018; Murayama-Hosokawa, et al., 2010; Swanton, et al., 2009; Walther et al. 2008; Lee, et al., 2011; Hieronymus, et al., 2018, https://doi.org/10.7554/eLife.37294). For example, Smid et al. (2011) found that elevated CIN and consequent high aneuploidy burden is associated with poor breast cancer prognosis, measured as time to distant metastasis. Carter et al. (2006) revealed a correlation between a transcriptional signature of CIN with metastasis, tumor grading, and clinical outcome in multiple human cancers. Paradoxically, some studies suggest that excessive levels of CIN negatively impact tumor fitness and associate with better survival outcome, possibly because too much chromosomal segregation errors can impart a number of cellular burdens that produce proinflammatory signals and lead to programmed cell apoptosis (Birkbak, et al., 2011; Jamal-Hanjani, et al., 2015; Tijhuis et al. 2019; Zasadil, et al., 2014). Given the widespread nature and far-reaching consequences of CIN in human cancer, strategies for targeting CIN as a therapeutic vulnerability in some cancers are being actively researched (Zasadil, et al., 2014) (Pierssens, et al., 2017). Despite its clear importance, CIN status is not readily testable for patients with cancer in routine clinical settings because it requires complicated experimental assessment involving live microscopy, sensitive detection of micronuclei (a consequence of CIN) via immunohistochemistry, or comprehensive genomic analysis. On the other hand, gold-standard histopathological examinations that are used for cancer diagnosis and grading are ubiquitously available. Here we sought to investigate the feasibility of using histopathology whole-slide images (WSIs) to predict CIN status.

Deep learning is a state-of-the-art methodology for analyzing and interpreting cancer histology images. In recent years, a large number of studies attempted to employ a deep learning approach for a variety of tasks in computational pathology field by taking advantage of deep learning's ability to extract hierarchical features from images in a direct and automatic fashion. Previous research has shown that presence of driver mutations, mutational signatures, and expression-defined tumor subtypes can be predicted from hematoxylin and eosin (H&E) slides (Coudray, et al., 2018; Schaumberg et al. 2016; Xu, et al., 2019; Chen, et al., 2020; Liao, et al., 2020). For example, Kather et al. (2019) trained a Convolutional Neural Network (CNN) model that can robustly predict genome microsatellite instability in gastrointestinal cancer from H&E histology, obtaining a patient-level area under the curve (AUC) of 0.84. Coudray et al. (2018) trained a deep learning network that successfully predicted six of ten most commonly mutated genes from lung adenocarcinoma pathology images, with AUCs from 0.733 to 0.856. Kather et al. (2020) then demonstrated the ability of deep learning to predict point mutations, molecular tumor subtypes, and immune-related gene expression signatures directly from H&E images in multiple cancer types. Fu et al. (Fu, Jung and TORNE 2020) used transfer learning and correlated histopathological pattern features with genomic, transcriptomic, and survival data in 28 cancer types. Javad et al. systematically used CNNs on 23 cancer types for tasks including tumor versus normal and cancer subtype classifications as well as predicting the presence of TP53 mutations (Noorbakhsh, et al., 2020).

In this study, we proposed using pathology images to predict patients' CIN status in breast cancer. We created a framework that uses transfer learning and feature aggregation to accurately discriminate high-CIN and low-CIN histopathology slides without human intervention. This framework is not limited to breast cancer and can be potentially extended to other cancer types. Our results indicate that (1) CIN can be predicted accurately from histopathology slides and (2) unexpectedly there appears to be substantial intra-tumor heterogeneity CIN status in many patients. These results pave the way for using CIN as a biomarker of prognosis and response to anti-CIN therapies fully integrated into existing clinical pathology workflows.

## Results

### A weakly supervised deep learning model for patient genomic CIN classification in breast cancer

**Figure 1:**
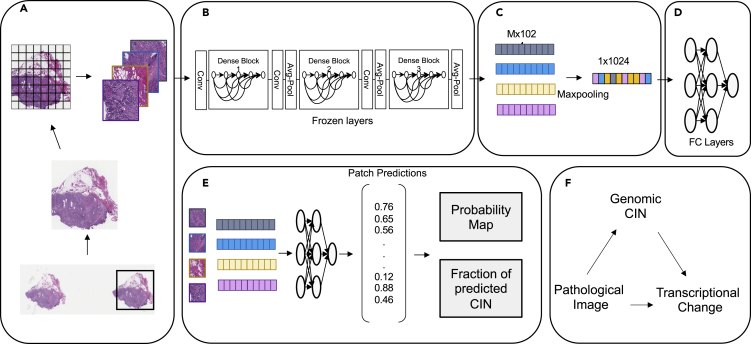
Overview of the pipeline (A) During mage preprocessing, an overall region of interest (ROI) was identified in WSI as window with the highest tissue percentage using a sliding window approach. Then the ROI was tiled into non-overlapping patches before quality control. Only qualified patches were kept as described in transparent methods. (B) Patch-level feature extraction was performed using a pre-trained CNN architecture. (C) Max-pooling layer was used for aggregating patch-level feature embeddings to patient-level feature embedding. (D) Fully connected layers were trained in supervised approach based on patient-level genomic CIN status. (E) Patch-level feature embeddings were fed into trained fully connected layers from (D). Probability maps based on patch predictions were generated, and fractions of predicted CIN that measure slide intra-tumor heterogeneities were calculated. (F) Pathological images were used to predict genomic CIN. Since genomic CIN can potentially alter gene expression and pathways on the transcriptional level, differentially expressed gene analysis and pathway analysis were performed. Pathology-predicted CIN and Genomic CIN were compared with CIN transcriptional signatures.

We obtained H&E slides and genomic profiles from patients with breast cancer in TCGA. Here we quantify CIN using the fraction genome altered (FGA, see transparent methods), which is one of the most commonly used quantitative measurements of CIN (Sipos, et al., 2021; Burrell, et al., 2013). FGA quantifies the burden of aneuploidies detectable in bulk genomic profiles. Although FGA is not a perfect measure of CIN and is incapable of capturing the rate of chromosomal changes by only providing a snapshot of the chromosomal alteration state, we and others have observed a strong correlation between FGA and CIN measured using microscopy and/or micronuclei staining (Schonhoft, et al., 2020; Pikor, et al., 2013). We refer to FGA as genomic CIN score, to contrast it with pathology predicted CIN scores introduced in this study. Genomic CIN score higher than 0.3 was labeled as high CIN; genomic CIN score lower than 0.3 was labeled as low CIN (Figure S1). H&E slides were processed as described in transparent methods. Based on our CIN classification, the breast cancer (BRCA) cohort had 485 high-CIN patients with 515 WSIs and 23,427 patches, and 525 low-CIN patients with 550 WSIs and 23,568 patches (Table S1). This study presents a deep learning model that can automatically predict patients' genomic CIN status on the molecular level from H&E-stained histopathology slides (Figures 1A–1E, see transparent methods). Our model uses CNN models pre-trained on ImageNet as patch-level feature extractor (Figure 1B) and then aggregates patch features into patient features (Figure 1C). This approach not only effectively addresses intra-tumor heterogeneity using “weak” patient-level labels but also offers opportunity to explore spatial CIN heterogeneity in individual patients (Figures 1D and 1E). The 1,070 WSIs of 1,010 patients from TCGA-BRCA were randomly split into training, validation, and test set. We then evaluated model performance in the hold-out test set, which included 152 patients.

### Deep learning model predicts CIN with high accuracy and sensitivity

**Figure 2:**
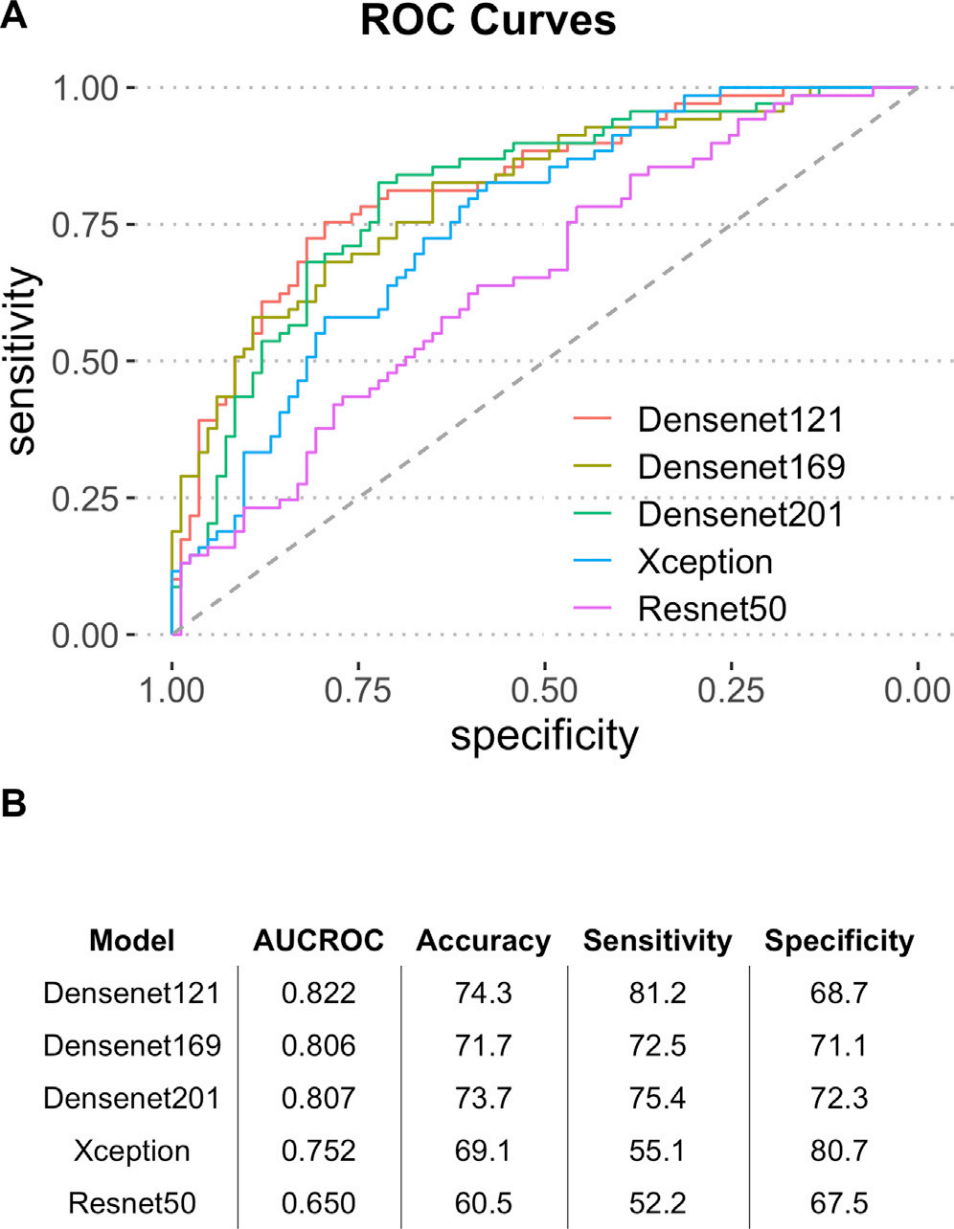
Model performance final evaluation Model performance was evaluated in test set. (A) Receiver operating characteristic (ROC) curves for different CNN architectures. (B) Table for model performance with AUC, accuracy, sensitivity, and specificity.

Several commonly used CNN architectures were tested in the transfer learning step used to extract the most relevant features that can predict genomic CIN. The best feature extraction method was selected based on the ability of trained fully connected layers to predict CIN groups in validation dataset (Figure S3). Results shown in Figure 2 indicate that Densenet-121 achieved the best performance with an AUC of 0.822 and accuracy (ACC) of 74.3%. Densenet networks with different depths achieved similar performance with AUCs of 0.806 and 0.807 for Densenet-169 and Densenet-201, respectively. The Densenet-121 model got a good sensitivity of 81.16%. The Xception model achieved an AUC of 0.752 and Resnet-50 achieved 0.650. Since the models were trained to classify genomic CIN status based on a hard threshold of genomic CIN score measured by FGA of being 0.3, we thus evaluated the model performance for patients with very high CIN (FGA>0.4) versus very low CIN (FGA<0.2) as well as patients who have moderate genomic CIN scores (FGA: 0.2–0.3 versus FGA: 0.3–0.4). Results shown in Table S2 indicate that, although our model performs best for predicting very low and very high CIN (ACC, 77.6%; AUC, 0.83), it retains good performances in the intermediate CIN range (ACC, 66.7%; AUC, 0.77). Altogether these results indicate that a deep learning model can accurately classify CIN status, achieving an AUC of 0.822 with 81.2% sensitivity and 68.7% specificity in an independent test set not used for training or parameter exploration.

### High-CIN patients exhibit more atypical mitosis events

**Figure 3:**
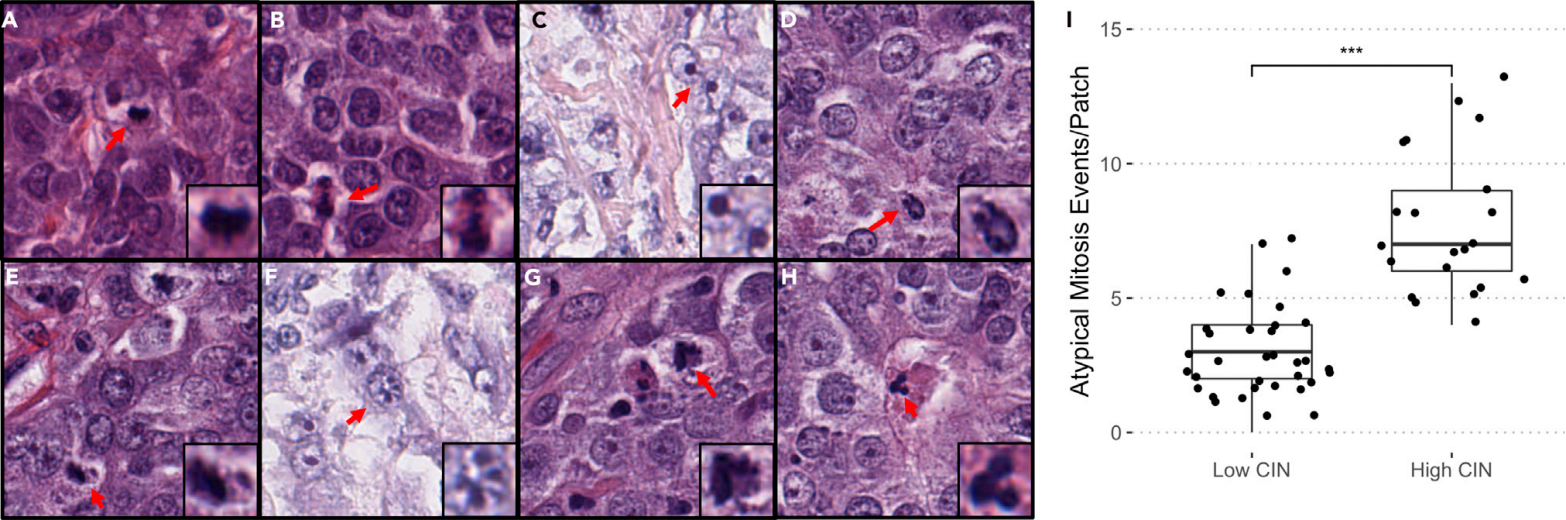
Patches containing normal and abnormal mitosis events at 40× magnification (A and B) Normal mitosis. (C)-(H) Abnormal mitosis. (C) Anaphase bridge. (D) Monopolar mitosis. (E–G) Mitotic figure with unaligned chromosomes. (H) Multipolar mitosis. (I) Boxplot of atypical mitosis events per field of view between five predicted high-CIN and five predicted low-CIN patients (GEE model p value<0.0001 ∗∗∗).

To independently validate that pathology-predicted CIN status (and genomic CIN) correlates with CIN-related aberrant mitotic events, we inspected 10 tumor slides at 40× magnification level, looking manually for aberrant mitotic events. Half of the 10 slides were predicted as high CIN and half as low CIN. All 10 slides were also concordantly labeled as high CIN or low CIN by genomic CIN (in other words, they were true-positive and true-negative predictions). The expert who conducted the inspections was not informed of the sample labels to reduce subtle bias. As shown in Figures 3A–3H, both normal and abnormal mitosis events, including anaphase bridge, spindles with misalignment chromosomes, and multipolar and monopolar chromosome arrangements, were observed. By fitting the generalized estimating equation (GEE) Poisson regression model, we found that atypical mitosis event counts per field of view (patches with fixed size of 1,024∗1,024 pixels on 40×) are significantly higher in predicted high-CIN patients compared with predicted low-CIN patients (GEE model p value<0.0001, Figure 3I).

### Patch predictions demonstrate intra-tumor heterogeneity

**Figure 4:**
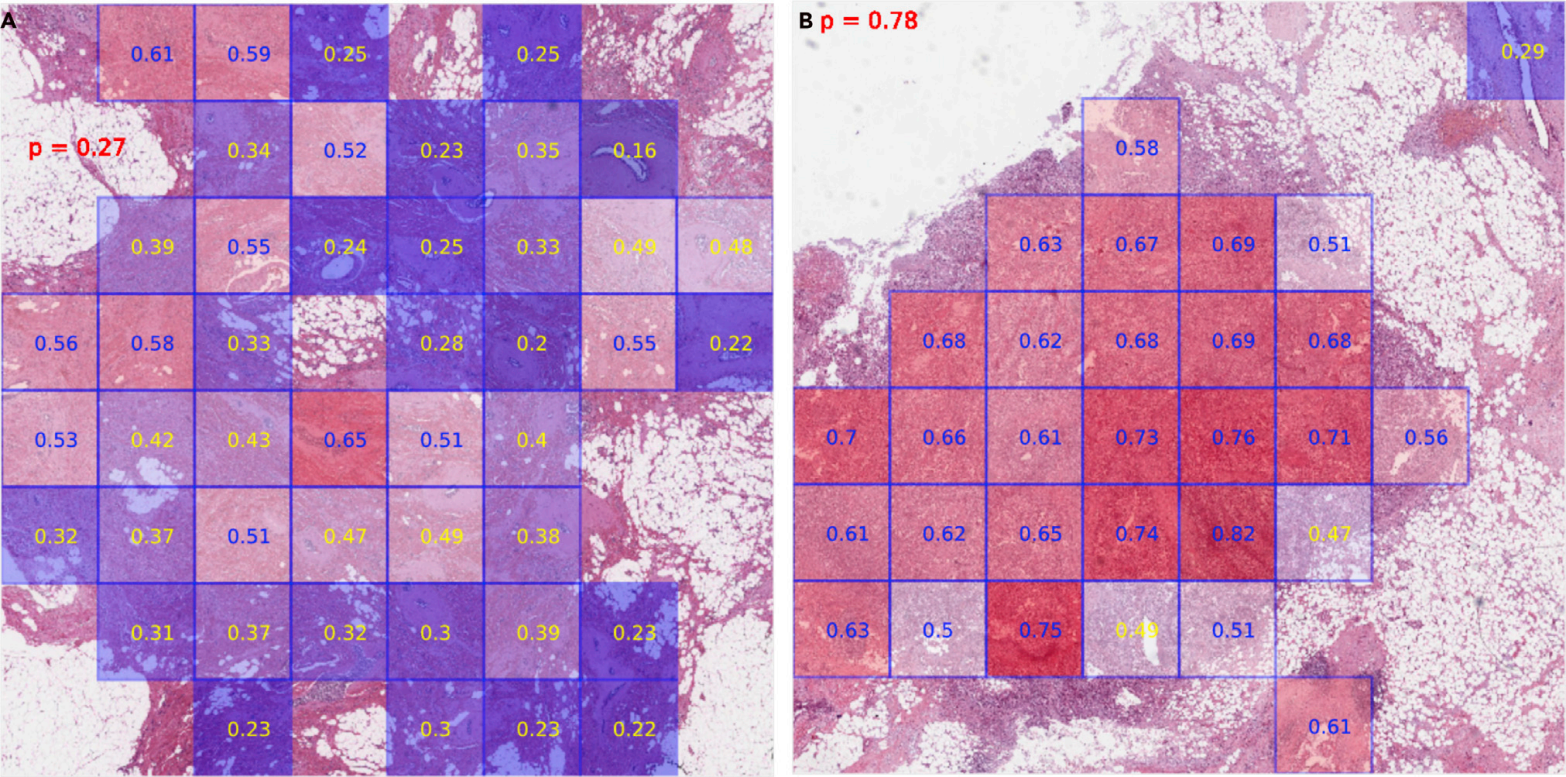
Intra-tumor heterogeneity by patch predictions Red shows predicted probability of high-CIN slide based on our deep learning model. Numbers within each grid imply patch-level predictions. Blue indicates high-CIN patch; yellow indicates low CIN patch. (A) An example of low-CIN slide in test set. (B) An example of high-CIN slide in test set

As discussed in the previous section, we had hypothesized that not all patches within the same slide may have the same level of CIN; in other words, we hypothesized that there may be intra-tumor heterogeneity in CIN within the same tissue section. To test this hypothesis, we visualized patch predictions within one WSI. Patch predictions were generated by feeding individual patch features into trained fully connected layers independently. As shown in Figure 4, both high-CIN and low-CIN patches can be found within one WSI regardless of the slide's CIN status. Figure 4A is an example of a low-CIN slide with predicted high-CIN probability of 0.27. Of all 45 patches, 11 (= 24.44%) were predicted as high-CIN patches. Because high-CIN patches still exist in low-CIN slides owing to intra-tumor heterogeneity, the prevalence scale and patch probability also have influence of the whole-slide CIN status. Similar results were shown in Figure 4B, where low-CIN patches were also found in high-CIN slide. We thus define predicted CIN-high fraction score as the percentage of predicted high-CIN patches based on each pathology image. Altogether we found that only 94 (9.31%) of 1,010 patients in the whole cohort exhibited a low level of intra-tumor heterogeneity with predicted CIN-high fraction score smaller than 10%. The median predicted CIN-high fraction score of this cohort is 57% with 25^th^ and 75^th^ percentile of 32% and 83%, respectively (Figure S2). A growing number of investigations have suggested the positive correlation between CIN status and increased neoplastic nuclear size, which can potentially be used as an excellent surrogate marker for CIN detection (Thompson and McManus 2015; Penner-Goeke, et al., 2017; Baergen, et al., 2019). Increases in DNA content (i.e., ploidy) have been found to be one of the mechanisms behind the corresponding nuclear enlargement (Zeimet, et al., 2011; Petersen, et al., 2009). To further validate the existence of intra-tumor heterogeneity revealed by patch predictions, we conducted nuclear instance segmentation and classification on all patches from test dataset using pretrained HoVer-Net model. Segmentation results showed that predicted CIN-High patches have larger neoplastic cell nucleus comparing with predicted CIN-Low patches (p value<0.0001, Figure S5A). At the same time, slides with a higher fraction of predicted CIN-High patches showed higher tumor ploidy values (p value: 0.0031, Figure S5B).

### Fraction of predicted CIN high patches is correlated with transcriptional CIN score

CIN, as a hallmark of cancer, has been linked to activation of key downstream biological pathways such as cGAS-STING and non-canonical nuclear factor κB (NF-κB) (Bakhoum and Cantley 2018). To bridge the gap between molecular genome alterations with pathological features (Figure 1F), we conducted correlation analysis between a CIN-driven transcriptional gene signature with both genomic CIN score and predicted CIN-high fraction score. CIN23 is a gene signature derived from the human metastatic cell line models (MDA-MB-231) engineered to over-express MCAK (to suppress CIN) or a dominant negative version of MCAK (to increase CIN) (Bakhom, 2018, Cailleau et al., 1978). Employing CIN23, we derived a gene signature score for each patient as transcriptional CIN score using ssGSEA. Genomic CIN score was positively correlated with transcriptional CIN score with correlation coefficient of 0.14 (Table S5, p value<0.0001). We then reasoned that the average predicted CIN intra-tumor heterogeneity measured by the percentage of predicted high-CIN patches of each slide may correlate with the transcriptional CIN score, which is also an average representation of CIN across all spatial areas. Indeed, we observed a weak but significant positive correlation with transcriptional CIN (Table S5, rho: 0.1, p value = 0.0026).

### Model performance is not breast cancer subtype specific

In the past, different patterns of CIN were observed to be associated with distinct subtypes of breast cancer (Kwei, et al., 2010). In this cohort, we found significant positive association between genomic CIN status with the prevalence of ER- (p value<0.0001), PR- (p value<0.0001), and Triple Negative (p value<0.0001) subtype status but not HER2 (p value = 0.1) status (Table S3). We therefore sought to verify that our algorithm is not simply predicting tumor subtypes (since we have that predicting breast cancer subtypes is feasible from H&E slides [Khosravi, et al., 2018]). We combined subtype information (ER, PR, HER2 status) along with clinical features including age, race, menopause status, and number of positive lymph nodes as clinical input. Then we retrained the fully connected layers with three different settings of input that are clinical input alone, image features alone, and concatenating clinical input with image, respectively. Results showed adding image features to clinical features can significantly help improve model performance (p value = 0.047). But when using image for predictions, adding extra clinical features along with subtype information did not improve the model performance (p value = 0.82) (Figure S4). Finally, no evidence suggested our model to be subtype specific with AUCs statistically the same across different subtypes (p values: ER, 0.33; PR, 0.94; HER2, 0.41; Triple Negative, 0.86) in this TCGA cohort (Table S4). We concluded that our model is predictive of CIN independently of tumor subtypes.

### CIN is associated with poor prognosis in breast cancer

**Figure 5:**
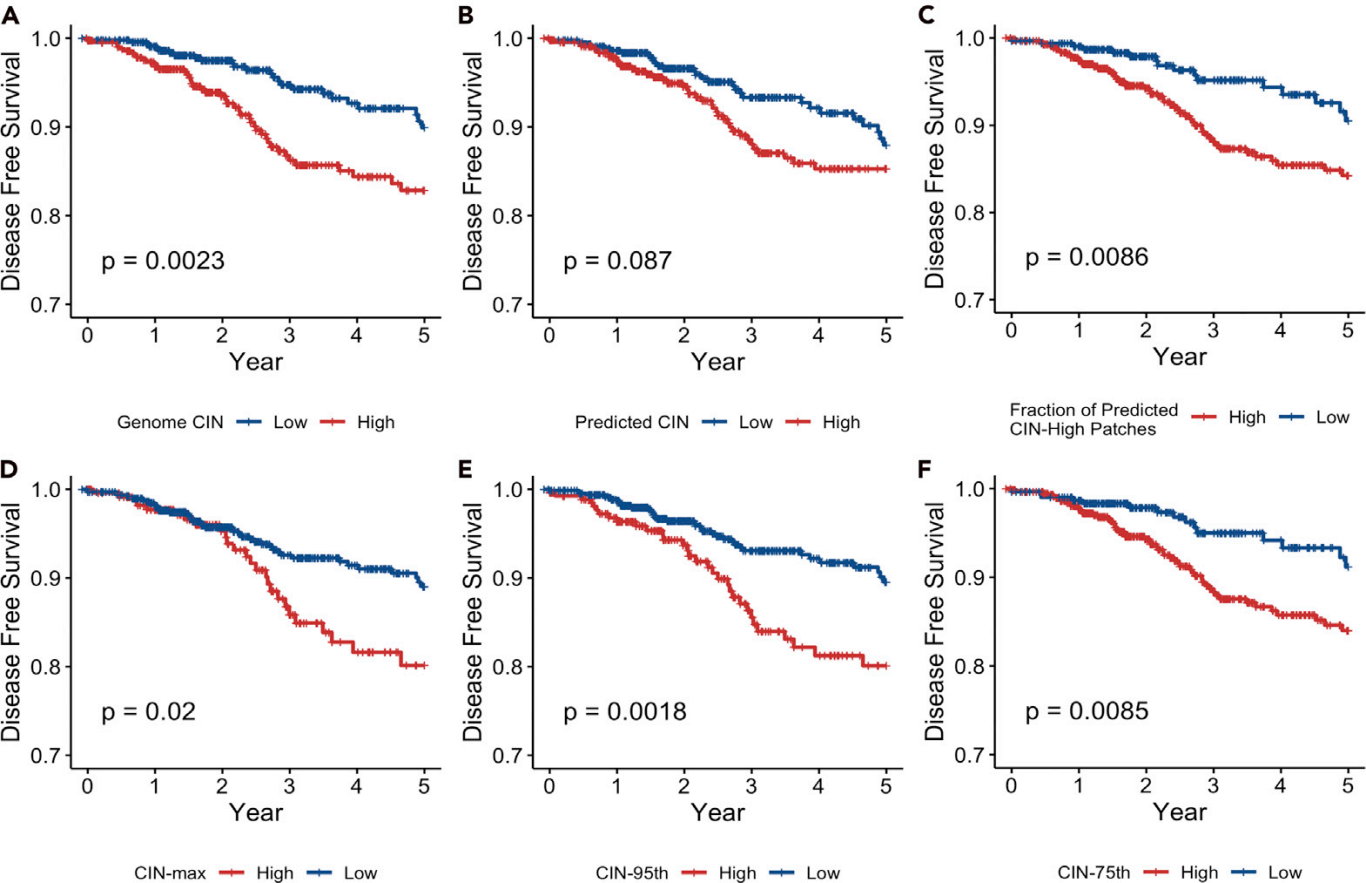
Kaplan-Meier curves of disease-free survival probabilities grouped by different CIN biomarkers p values were calculated by log rank test. (A) Genomic CIN (Stratification cutoff: 0.3). (B) Predicted CIN (Stratification cutoff: 0.44). (C) Fraction of predicted CIN high patches (Stratification cutoff: 0.42). (D) CIN-max, the maximum patch prediction within each slide (Stratification cutoff: 0.77). (E) CIN-95^th^, the 95^th^ percentile patch prediction within each slide (Stratification cutoff: 0.72). (F) CIN-75^th^, the 75th percentile patch prediction within each slide (Stratification cutoff: 0.55).

The association between CIN and cancer prognosis is complex and paradoxical. Some studies showed association of CIN with poorer cancer prognosis (Orsetti, et al., 2014; Carter, et al., 2006), whereas other studies reached opposite conclusions, suggesting that excessive level of CIN would suppress tumor progression and lead to better clinical outcomes (Birkbak, et al., 2011; Zasadil, et al., 2016). To further investigate this point, we conducted a survival analysis in the TCGA cohort aiming to explore the prognostic values of different CIN scores. In these analyses, we used maximally selected rank statistics to determine optimal prognostic CIN score cutoffs and log rank tests to evaluate the differences between survival curves. We found that high genomic CIN is associated with poorer 5 years' prognosis compared with low genomic CIN, where prognosis is measured as time to any events including new tumors or mortality (Figure 5A; p value = 0.0023). Pathology-predicted CIN using our deep learning model was also predictive of outcomes (Figure 5B; p value = 0.0045). Finally, the predicted CIN-High fraction score was also correlated with worse outcomes (Figure 5C; p value = 0.0086). We postulated that the presence of patches with high predicted CIN scores within each slide may be sufficient to impact clinical outcomes. We calculated different percentile CIN scores based on all patches of each slide (75^th^, 95^th^, and maximum). We found that all slide-level percentile CIN scores were prognostic (Figures 5D–5F; p values: CIN-75^th^, 0.0085; CIN-95^th^, 0.0018; CIN-max, 0.02) with CIN-95^th^ being the most robust prognostic score, even more predictive than genomic CIN. That CIN-95^th^ is more predictive than CIN-max can be explained by the lack of stability of maximal value and possibly by the need for more than one patches to have high CIN to impact outcomes.

### CIN is associated with profound transcriptional changes in tumor samples

We reasoned that our ability to predict CIN based on histopathology slides may underlie a relatively profound difference in biological features between CIN low and CIN high tumors, which may influence cell morphology and tissue structure in H&E slides. We therefore conducted differentially expressed genes and gene set enrichment analysis between high CIN and low CIN samples. (Figure 1F) To minimize the confounding effect of cancer subtypes caused by the unbalanced subtype distributions across CIN groups, we adjusted tumor subtypes in the design matrix and tested differentially expressed genes for genomic CIN term. A total of 307 differentially expressed genes were identified (logFC>1, adjusted p value<0.05) between CIN low and CIN high tumors as shown in Figure S10A. Cell cycle and mitosis-related gene signatures were up-regulated significantly in high-CIN tumor samples (Figure S10B). This analysis thus confirms substantial biological differences between CIN high and CIN low tumors.

## Discussion

In this study we demonstrate for the first time the ability to predict CIN based on H&E slides. At present, it is challenging to capture the ongoing rate of chromosome mis-segregation to identify CIN in routine clinical setting since mitotic alterations are rare in H&E slides; other assays such as microscopy or micronuclei staining have not been deployed in the clinical setting. Here we demonstrated the ability of using histopathology slide images to predict CIN status of each patient and achieved high accuracy (=74.3%) and sensitivity (=81.2%). Of equal importance, our model indicates the existence of intra-tumor heterogeneity in CIN levels and revealed its association with poorer clinical outcomes. Further research, perhaps based on regional sequencing, is needed to further validate these findings. The substantial prognostic impact potentially exerted by spatial sub-regions (patches) with the highest CIN scores is important since it indicates that such regions may drive response to treatment. Future treatment modalities may need to focus on eliminating CIN high tumor cells if they are to achieve maximal therapeutic impact. Either way, our results pave the way for integrating CIN as prognosis biomarker and therapeutic vulnerability into existing clinical pathology settings.

One of the main challenges of computational pathology is to manage the trade-off between abundant morphological information and large size of WSI. Splitting WSI into hundreds of thousands of patches and training neural networks on the patch level is a commonly used strategy (Srinidhi et al. 2021). As mentioned above, there are several studies that successfully demonstrated the ability of using H&E-stained histology to predict genetic mutations using this patch-level learning approach (Khosravi, et al., 2018; Kather et al., 2019). We also experimented using patients' level labels to supervise patch learnings directly with the same approach but failed in predicting CIN levels. We reasoned there might exist substantial intra-tumor heterogeneity within individual slides and that therefore using patient-level CIN labels are not directly applicable to patches for training. To overcome this obstacle for CIN status learning, we applied a weakly supervised learning approach. More specifically, we used transfer learning to extract patch-level features followed by addition of a max-pooling layer with only maximum feature values kept along each feature dimension so that most irrelevant features that would potentially add noise to the model learning throughout the WSI were removed during this step. The features that were kept training the top fully connected layers were distributed widely throughout the WSI but not from a local region so that intra-tumor heterogeneity problem is reduced during training. We further compared model performance between slide predictions with patch predictions in predicting slide-level genomic CIN status to see the influence of this feature aggregation strategy. Contrasted with slide-level predictions that obtained an AUC of 0.82, patch-level predictions only got an AUC of 0.66 (Figure S11). This difference can be explained by patch-level predictions only representing predictions based on local morphological features, whereas slide predictions look at global morphological features. In addition, slide-level predictions exhibited a high correlation with continuous genomic CIN score (Figure S12, Spearman rho, 0.52; p value<0.0001), whereas only binary labels were available during the training. We successfully demonstrated the effectiveness of this strategy by achieving high accuracy (=74.3%) in classifying genomic CIN status in the test dataset.

Digital pathology images can be examined at different magnification levels. We experimented on both 2.5× and 10× magnified tiles for the predictions. We found that 2.5× achieved more accurate predictions marginally than 10× (2.5× AUC, 0.82; 10× AUC, 0.76; p value = 0.06) and multi-scaled model by combining 2.5× with 10× magnification features (2.5× AUC, 0.82; multi-scaled AUC, 0.81; p value = 0.59), although not statistically different tested by DeLong's method (Figure S4). We reasoned that each tile on the 2.5× level can capture more relevant features with a wider spatial view than high-resolution tiles, whereas on the 10× magnification level, tiles were more likely to be covered up by some “unknown” irrelevant features. A similar observation was made in Coudray's study (Coudray, et al., 2018) that analyzing 5× patches led to higher accuracy than 20× patches.

To validate the rationale of utilizing pathological slide images to infer genomic alterations, we performed differentially expressed gene analysis between CIN low and CIN high patients. The results revealed the impact of CIN in breast cancer including activating multiple pathways relating to cell cycle and mitosis (Figure S10B). As expected, mitotic alterations can be identified in slide images on high-resolution views. Future studies may focus on training machine learning models to detect aberrant mitotic events directly from H&E slides. This will require a very large training set of such events, which is currently not available.

### Limitations of the study

In this study we used a bulk genomic aneuploidy burden approach to approximate patients' CIN status. Aneuploidy burden is a good but imperfect proxy for CIN, in that it focuses on highly clonal events and does not capture ongoing CIN. Moreover, in the absence of multi-region genomic sequencing, it is not possible to get a regional genomic CIN estimation. This limits our ability to demonstrate that our approach can capture spatial heterogeneity in CIN status. Better measurement for CIN can provide more accurate labels for training deep learning models as well as validating patch-level predictions. In addition, our results showed models on 2.5× magnification performed better than 10× magnification. We speculate that patches on a lower magnification scale contain more spatial context compared with the ones with higher resolution of the same pixel sizes, thus explaining our results. On the other hand, using a lower resolution may miss important morphological feature differences including abnormal mitosis caused by CIN. Combining our low-resolution approach with sophisticated computer vision approaches that identify features such as aberrant mitotic events as assessed manually for a small number of image patches in our study may help improve CIN prediction accuracy in the future.

### Resource availability

#### Lead contact

Further information and requests for resources should be directed to and will be fulfilled by the Lead Contact, Dr. Olivier Elemento (ole2001@med.cornell.edu).

#### Materials availability

This study did not generate new unique reagents.

#### Data and code availability

All image and genetic data associated with this study can be downloaded from TCGA website (https://www.cancer.gov/about-nci/organization/ccg/research/structural-genomics/tcga). The source code and the guideline are publicly available at https://github.com/eipm/CIN.

## Methods

All methods can be found in the accompanying transparent methods supplemental file.

## Supplemental information

Document S1. Transparent methods, Figures S1–S12, and Tables S1–S5
