# Supplementary material for: Deep learning predicts chromosomal instability from histopathology images
Source: iScience. 2021 Apr 3;24(5):102394. doi: 10.1016/j.isci.2021.102394 (PMC8099498; doi:10.1016/j.isci.2021.102394)
Supplement: Document S1. Transparent methods, Figures S1–S12, and Tables S1–S5 [file mmc1.pdf]

iScience, Volume *24*

Supplemental information

Deep learning predicts chromosomal instability from histopathology images

Zhuoran Xu, Akanksha Verma, Uska Naveed, Samuel F. Bakhoum, Pegah Khosravi, and Olivier Elemento

Supplemental Figures and Tables

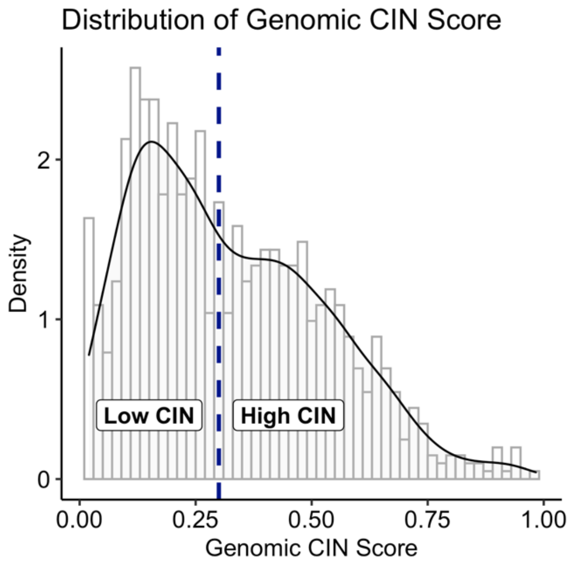

Figure S1. Distribution of Genomic CIN Score. Related to Figure 1. Fraction genome altered (FGA) was calculated to represent genome CIN score. Blue dash line indicates genomic CIN score of 0.3. Patients with genomic CIN score higher than 0.3 were classified as high CIN, otherwise as low CIN.

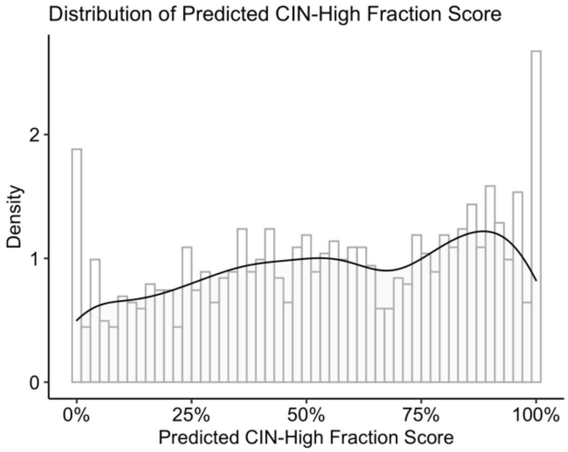

Figure S2. Histogram of Predicted CIN-High Fraction Score. Related to Figure 4. Predicted CIN-High fraction score is defined as the percentage of predicted high CIN patches based on each pathology slide image.

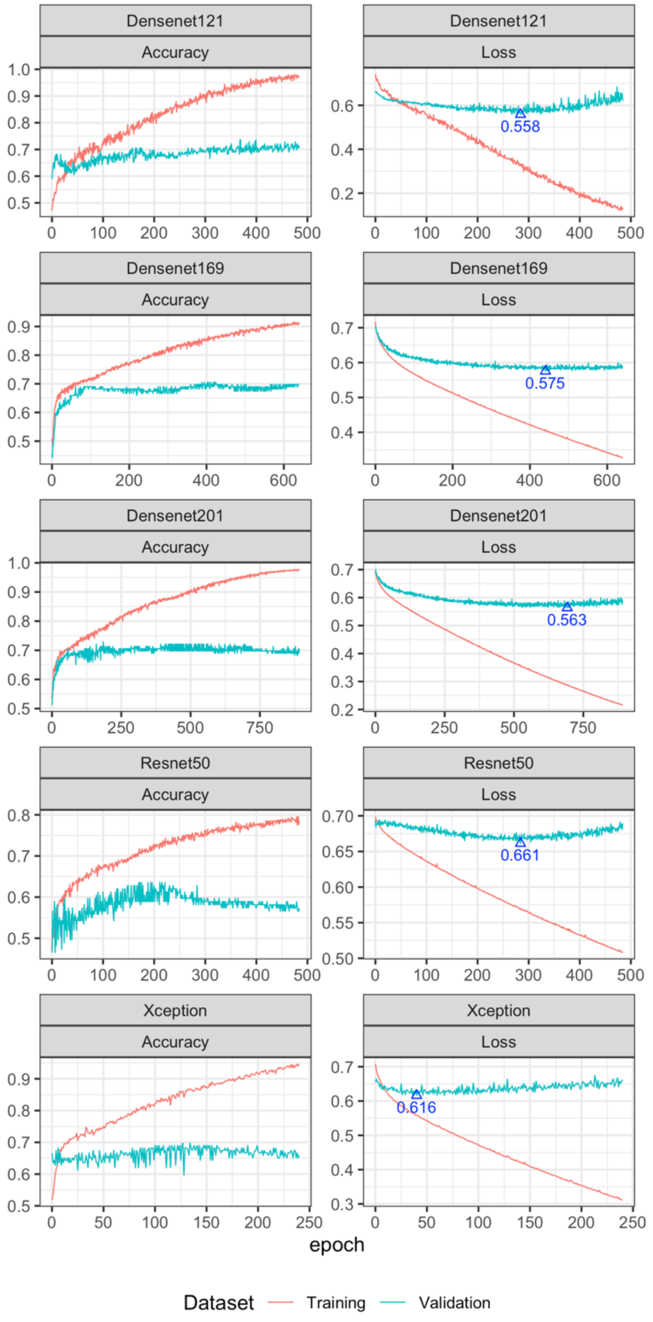

Figure S3. Model learning curves during training. Related to Figure 2. Triangle point implies the epoch with the lowest validation loss where models were chosen for further evaluations in blind test dataset.

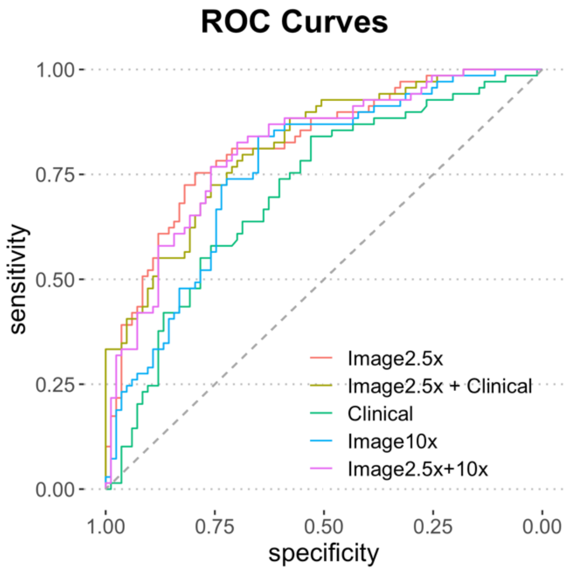

Figure S4. Receiver operation characteristic (ROC) curves of different model configurations. Related to Figure 2. Image 2.5x: AUC 0.82 (0.75, 0.89). Image 2.5x and clinical: AUC 0.82 (0.75, 0.89). Clinical variables only: AUC 0.71 (0.63, 0.79). Image 10x: AUC 0.76 (0.68, 0.84). Multiscale by combining 2.5x and 10x: AUC 0.81 (0.74, 0.88).

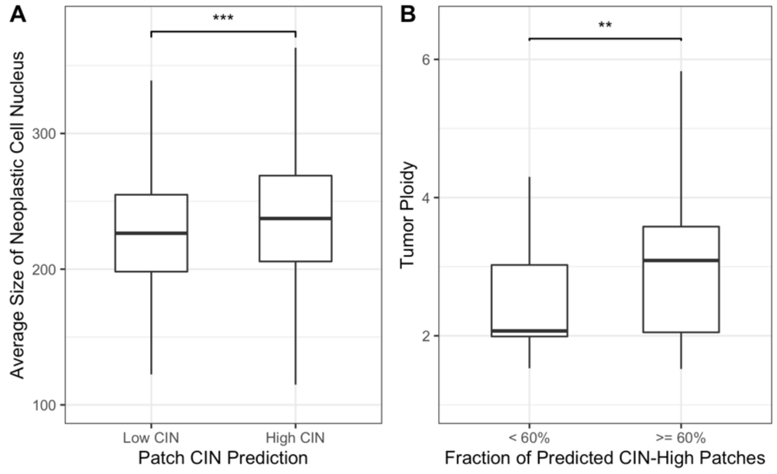

Figure S5. Boxplots of average size of neoplastic cell nucleus and tumor ploidy in test dataset. Related to Figure 4. (A) Boxplot of average size of neoplastic cell nucleus (pixels) between predicted low CIN patches and predicted high CIN patches. (Wilcoxon test, *** p-value<0.0001) **(B)** Tumor ploidy (patient level) between slides with low and high fraction of predicted CIN-High patches. Cutoff of 60% was chosen based on median value of fraction of predicted CIN-High patches in test dataset. (Wilcoxon test, ** p-value=0.0031)

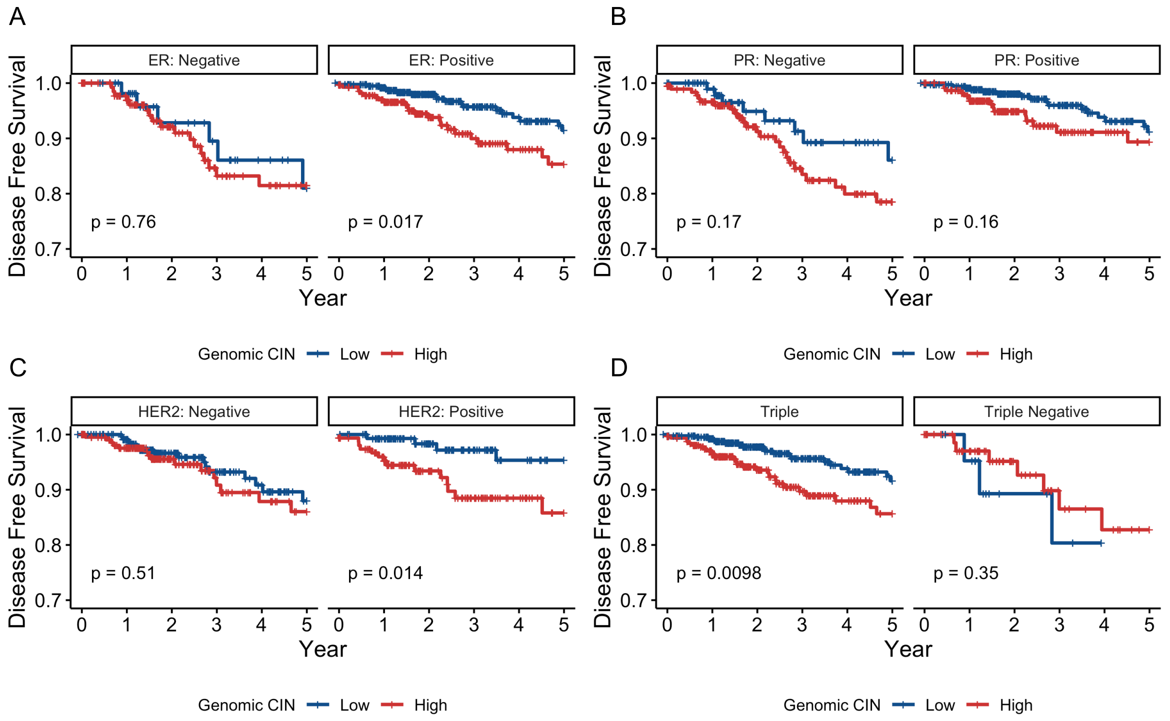

Figure S6. Kaplan-Meier curves of disease-free survival (DFS) probabilities by genomic CIN for different subtypes of breast cancer. Related to Figure 5. P-values were calculated by log rank test. Stratification cutoff: 0.3.

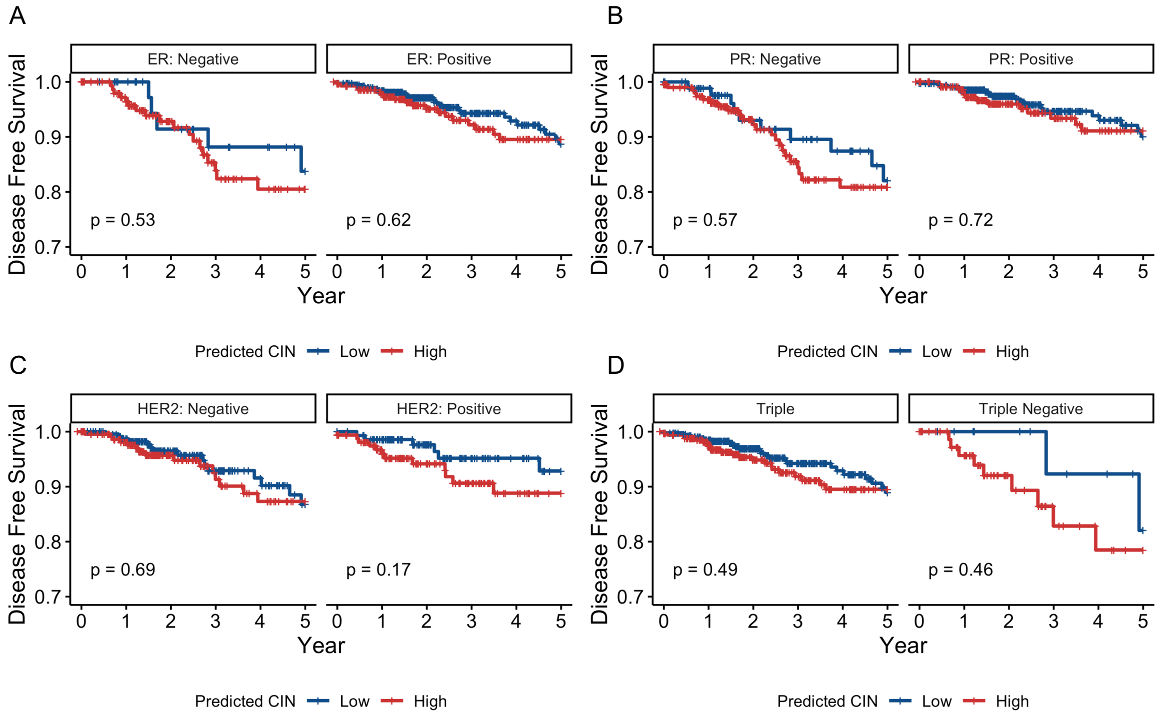

Figure S7. Kaplan-Meier curves of disease-free survival (DFS) probabilities by predicted CIN for different subtypes of breast cancer. Related to Figure 5. P-values were calculated by log rank test. Stratification cutoff: 0.44.

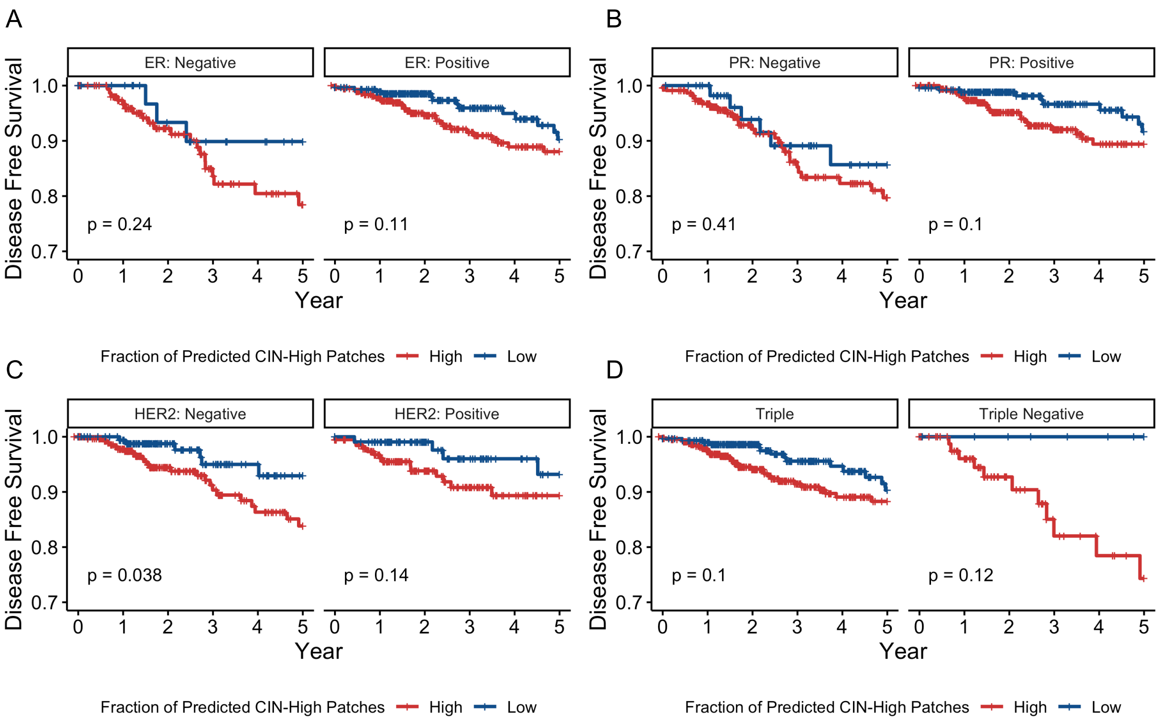

Figure S8. Kaplan-Meier curves of disease-free survival (DFS) probabilities by pathological CIN for different subtypes of breast cancer. Related to Figure 5. P-values were calculated by log rank test. Stratification cutoff: 0.42.

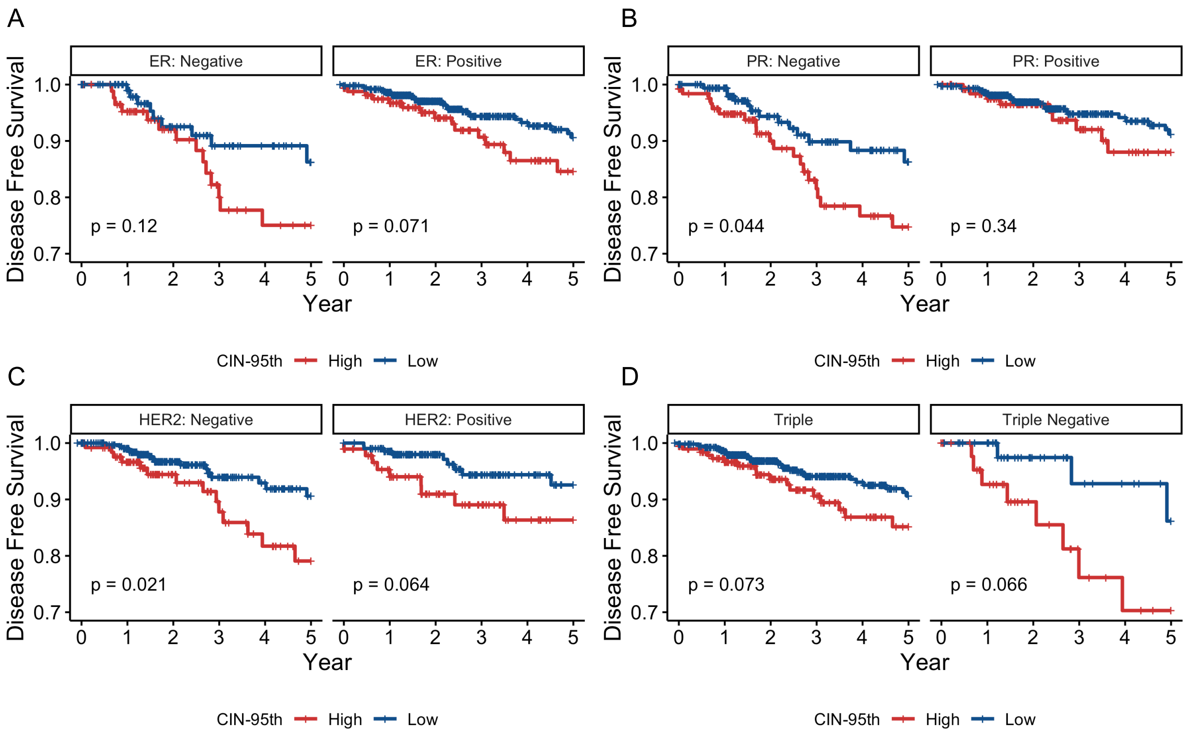

Figure S9. Kaplan-Meier curves of disease-free survival (DFS) probabilities by 95th percentile patch CIN for different subtypes of breast cancer. Related to Figure 5. P-values were calculated by log rank test. Stratification cutoff: 0.72.

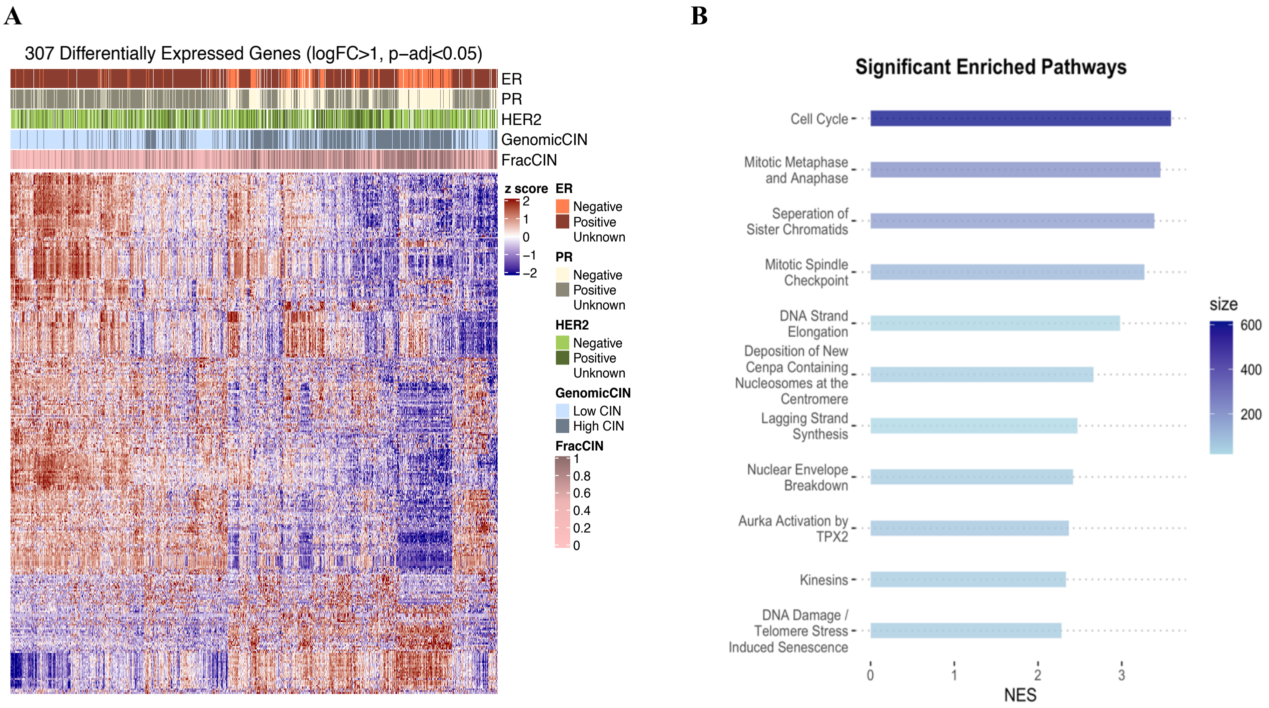

Figure S10. Differentially expressed genes and gene set enrichment analysis. Related to Figure 1F and Figure 3. (A) Heatmap of top differentially expressed genes between high CIN and low CIN with logFC>1 and ajudusted p<0.05. FracCIN represents predicted CIN-High Fraction. **(B)** Gene set enrichment analysis.

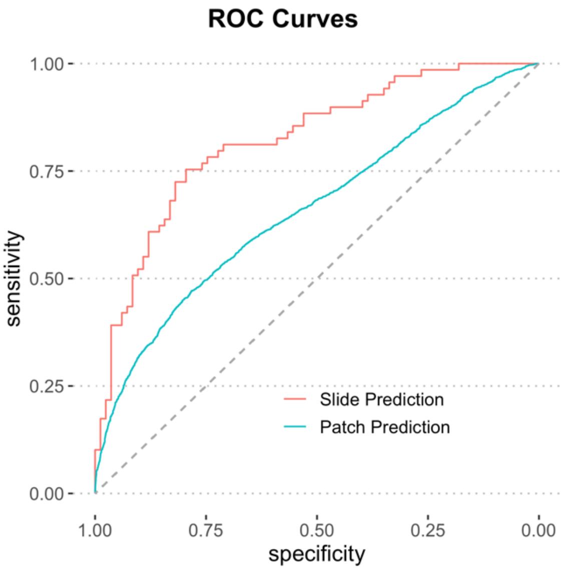

Figure S11. Receiver operation characteristic (ROC) curves for slide predictions and patch predictions from Densenet121 model. Related to Figure 2. Slide Prediction (Slide level prediction from aggregated slide level feature embedding): AUC 0.82 (0.75, 0.89). Patch Prediction (Patch level predictions evaluated by slide level labels): AUC 0.66 (0.65, 0.67)

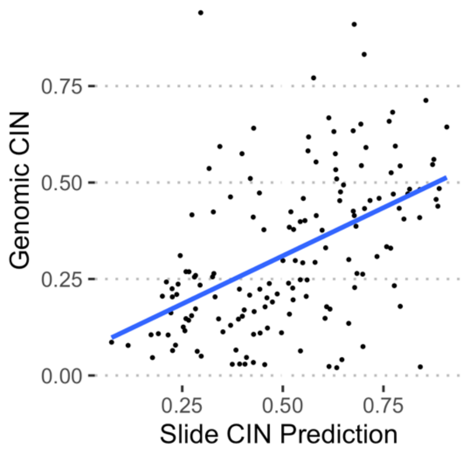

Figure S12. Scatter plots of slide CIN predictions with genomic CIN. Related to Figure 2. Blue lines represent regressed correlation lines. High CIN is defined by genomic CIN larger than 0.3. Spearman Correlation coefficient: 0.52, *** p-value<0.0001

Table S1. Patient and WSI numbers for training, validation and test set. Related to Figure 1 and Results. Statistics are in format of: Patient number (WSI number, Patch number). †: Total number of patients in training and validation set is 858, with further split into 730 for training (85%) and 128 (15%) for validation.

	Whole Dataset	Training and Validation Set	Test Set
High CIN	485 (515, 23427)	416 (441, 20128)	69 (74, 3299)
Low CIN	525 (550, 23568)	442 (463, 20045)	83 (87, 3523)
Total	1010 (1065, 46995)	858 (904, 40173) †	152 (161, 6822)

Table S2. Sample size and model performance in test dataset for very high CIN vs very low CIN and moderate high CIN vs moderate low CIN. Related to Figure 2.

	Comparison Samples	Test Sample Size	Accuracy	AUC-ROC
vs	<0.2 (very low CIN)	50	0.776	0.83
	>0.4 (very high CIN)	57		(0.75, 0.91)
vs	0.2-0.3 (moderate low CIN)	33	0.667	0.77
	0.3-0.4 (moderate high CIN)	12		(0.61, 0.93)

Table S3. Genomic CIN is associated with gene-expression subtypes of ER, PR and Triple Status. Related to Figure 2 and Results. Numbers in the table represent counts (percentage in each genome CIN group).

		Genomic CIN		p value
		Low	High	
ER	Positive	436 (87.0)	302 (65.4)	<0.0001
	Negative	65 (13.0)	160 (34.6)	
PR	Positive	387 (77.2)	251 (54.4)	<0.0001
	Negative	114 (22.8)	210 (45.6)	
HER2	Positive	158 (36.3)	171 (41.4)	0.1
	Negative	227 (63.7)	242 (58.6)	
Triple	Triple	467 (94.7)	353 (80.8)	<0.0001
	Tripe Negative	26 (5.27)	84 (19.2)	

Table S4. Model performance for subgroups based on molecular breast cancer types. Related to Figure 2, Results and Table S3.

		ROC	P
ER	Positive	0.79 (0.70, 0.87)	0.33
	Negative	0.87 (0.73, 1.00)	
PR	Positive	0.81 (0.73, 0.90)	0.94
	Negative	0.81 (0.67, 0.94)	
HER2	Positive	0.84 (0.71, 0.96)	0.41
	Negative	0.77 (0.66, 0.87)	
Triple	Triple	0.77 (0.68, 0.86)	0.86
	Tripe Negative	0.79 (0.57, 1)	
Overall		0.82 (0.75,0.89)	

Table S5. Correlations between transcriptional CIN score with genomic CIN score and Predicted CIN-High Fraction score. Related to Figure 1E, Figure 1F and Results. ER: Estrogen receptor. PR: Progesterone receptor. HER2: Human epidermal growth factor receptor 2.

	**Genomic CIN**		**Predicted CIN-High Fraction**	
	Correlation	P	Correlation	P
ER-	0.2005	0.0026	-0.0052	0.9379
ER+	0.0466	0.2079	0.0338	0.361
PR-	0.2157	0.0001	0.113	0.0425
PR+	0.0237	0.5524	-0.0021	0.9572
HER2-	0.1838	<0.0001	0.1495	0.0007
HER2+	0.0172	0.756	0.0182	0.7422
Non Triple	0.0634	0.0704	0.0463	0.1869
Triple-	0.1769	0.0659	-0.0464	0.6322
Overall	0.1403	<0.0001	0.0952	0.0026

Transparent Methods:

Dataset:

Whole slide images along with clinical and genomic data were downloaded from The Cancer Genome Atlas (TCGA), project TCGA-BRCA using the TCGAbiolinks R package (Colaprico, et al. 2015). Only formalin-fixed paraffin-embedded (FFPE) diagnostic H&E-stained histopathology slides from primary tumor sites were used for this study. After removing slides that lack magnification information, and/or slides with artefacts including tissue folding, air bubbles and out-of-focus regions, the cohort consisted of 1,010 patients with 1,070 whole slide images (WSI).

Fraction genome altered (FGA), which was defined as the ratio of *Sum of altered genome size/ Total genome size analyzed*, was calculated using whole exome sequencing based copy number variation (CNV) data on the same TCGA patients. Copy-number segments were downloaded from TCGA. Segments with log transformed mean copy number values larger than 0.2 or less than -0.2 (Salas, et al. 2017, Ali Hassan and Mokhtar 2014) were treated as altered segments, respectively. Based on examining the FGA distribution for all 1,010, patients, we labelled patients with FGA less than 0.3 as low CIN; those with FGA above 0.3 were labelled as high CIN.

Image Preprocessing:

First, a single overall region of interest (ROI) with dimension of 2,048x2,048 pixels on 2.5x magnification (4mpp) was determined by a sliding window approach from each whole slide image, which typically has dimension of about 8,000x4,500 pixels. A simple thresholding method was used to distinguish tissue from white space background on greyscale space. All pixels with value lower than 215 were treated as tissue, otherwise as background. The ROI window that contained the highest percentage of tissue was kept for further processing. The selected window was then split into 8x8 non-overlapping patches each with dimension of 256x256 pixels. Quality control on patch level was conducted using the following method. All patches with tissue percentage less than 80% or with significant blurriness, pen marks or folded tissues were deleted. Color normalization was then performed to reduce batch effects across different data sources (**Figure 1A**). We performed Reinhard normalization to transform the color characteristics to a desired standard defined by the mean and standard deviations of target image (TCGA-AN-A0FK) from the cohort using Python library of HistomicsTK (Gutman, et al. 2017). Patients with more than one WSIs were treated as having one big WSI and can get more than 64 (8x8) patches depending on how

many WSIs they have, but 64 qualified patches were randomly chosen to prevent over-representing those patients. Overall, after deleting all unqualified patches, we obtained median of 51 (IQR: 32, 61) qualified patches for each patient that to be used for transfer learning.

Transfer Learning:

(1) Feature Extraction:

Instead of training CNN architectures from scratch, we used commonly used pre-trained models as feature extractors. We passed all patches (256x256 pixels) of each patient through Densenet-121, Densenet-169, Densenet-201 (Huang, et al. 2017, DOI: 10.1109/CVPR.2017.243), Xception (Chollet 2017) and Resnet-50 (He, Zhang, et al., Deep Residual Learning for Image Recognition 2016, DOI: 10.1109/CVPR.2016.90) networks that were pre-trained on ImageNet (Deng, et al. 2009) without top layers. Then we got a set of feature vectors for every patient with dimensions of m*n where m denotes number of patches of one patient and n implies number of features according to specific architecture that was used. For example, we got matrix of 32*1,024 for patch features with the patient who has 32 patches by using Densenet-121. To adapt patch level features to patient level labels and reduce the noise generated by intra-tumor heterogeneity, we applied a max-pooling layer on top of patch features and got patient level features with the same dimension for all patients (**Figure 1B**) (Courtiol, et al. 2018).

(2) Train Fully Connected Layers:

The whole cohort was randomly divided into training and validation set (n=858 patients, 85%) and hold-out testing set (n=152, 15%) without any overlap for both patients and images. Then the 858 patients were further split into training (730, 85%) and validation (128, 15%) set for tuning hyperparameters. We implemented several fully connected layers to take patient level features extracted by each of the CNN architectures mentioned above as input respectively (**Figure 1C**). Therefore, output by each model will be a prediction probability of high CIN class patient. Our model used binary cross-entropy loss function. We initialized the weights of fully connected layers with He initialization (He, Zhang, et al., Delving Deep into Rectifiers: Surpassing Human-Level Performance on ImageNet Classification 2015). Adam optimizer was used for the training network weights with learning rate of 0.00001. Training was stopped early (Prechelt 2012) if validation loss was not improving within 200 epochs. Epoch numbers were selected as per lowest validation

loss. Then model performance metrics including ROC curve and AUC, balanced accuracy, sensitivity and specificity were calculated in the hold-out test set for the final evaluation (**Figure 1D**).

Visualization of predicted patches:

The trained fully connected layers from last step can be fed with both combined patches (patient level features) and individual patches (patch level features) for hierarchical predictions. We predicted both patient level and patch level high CIN probabilities and visualized the predictions to demonstrate the existence of intra-tumor heterogeneity (**Figure 1E**).

Pathological, Genomic and Transcriptomic scoring of CIN:

We define predicted CIN-high fraction score as the percentage of predicted high CIN patches based on each pathology slide image. CIN-max, CIN-95th and CIN-75th are defined as maximum, 95th percentile and 75th percentile of patch predictions within each slide, respectively. Genomic CIN score was calculated by fraction genome altered as mentioned earlier (Ali Hassan and Mokhtar 2014). CIN23 gene signature (Bakhom 2018) score was computed using single sample Gene Set Enrichment Analysis (ssGSEA) to indicate transcriptional CIN score from RNA expression data of the same cohort.

Transcriptome analysis:

We examined differentially expressed genes of primary tumor between high CIN and low CIN patients using Limma R package (Ritchie, et al. 2015). Differential expressed genes and samples shown in heatmap were clustered using Euclidean distance. Gene set enrichment analysis was conducted using fgsea (Korotkevich, et al. 2019, DOI: https://doi.org/10.1101/060012) R package and Reactome pathway database (https://reactome.org).

Mitosis events inspection:

Among all the patients who have agreed predicted CIN and genomic CIN status, top five extreme high CIN and low CIN patients were selected according to genomic CIN score. Several tumor patches of one patient with dimension of 1,024x1,024 on 40x magnification were randomly inspected and atypical mitosis events number in each patch were recorded.

Nuclear segmentation and Tumor ploidy:

We performed nuclear instance segmentation for all the patches from test dataset and classified the nuclei into five categories (neoplastic cell, non-neoplastic epithelia, inflammatory cell, connective cell and dead cell) automatically using Hover-Net model (Graham, et al. 2019) which was pretrained in PanNuke dataset (Gamper, Koohbanani and Benet, et al. 2019, Gamper, Koohbanani and Benes, et al. 2020) that consists of 205,343 labeled nuclei from 19 different tissue types. The average size of neoplastic cell nucleus was calculated for each patch using the total number of pixels of neoplastic cell nuclei divided by the instance number of neoplastic cell nuclei. Tumor ploidy data in which the estimations were obtained from ABSOLUTE (Carter, et al. 2012) was downloaded from Pan-Cancer Atlas (https://gdc.cancer.gov/about-data/publications/pancanatlas) (Weinstein, et al. 2013).

Statistical analysis and Software:

Training of our DNN method was performed on local computer powered by one NVIDIA GeForce GT 640M GPU with 512 MB memory and one 2.7-GHz Quad-Core Intel Core i5 CPU. All statistical and bioinformatics analyses were performed in R, version 3.6.2. Image preprocessing and neural network training were conducted in Python, version 3.7.4. ROC curves were compared using DeLong's method by R package of pROC. Chi-square test was conducted to test the independency between cancer subtypes with genomic CIN status. Spearman's rank-order correlation test was performed for the correlation analysis without distribution assumption. Wilconxon rank sum test was used to compare the average size of neoplastic cell nucleus between high CIN and low CIN patches as well as tumor ploidy between slides with high and low fraction of predicted CIN-High patches. Log rank test was used for comparing Kaplan-Meier survival curves between different genome and predicted CIN groups. Time to any new tumor events and mortality was used as composite survival events and the data was censored at 5 years. Maximally selected rank statistics (Lausen, et al. 2004, Hothorn and Lausen 2003) was used to determine the optimal prognostic cutoff points for CIN biomarkers including predicted CIN, predicted CIN-High fraction, CIN-max, CIN-95th and CIN-75th. Generalized estimating equation (GEE) of Poisson regression model was used to compare atypical mitosis event number between CIN high and CIN low groups. All statistical tests were two sided with $p<0.05$ indicated significant. OpenSlide

python was used for reading and tiling whole-slide images. TensorFlow2 were used for building and training neural networks.

Supplemental Reference

Ali Hassan, Nur Zarina, and Norfilza Mohd Mokhtar. 2014. "Integrated analysis of copy number variation and genome-wide expression profiling in colorectal cancer tissues." *Plos One* 9 (4).

Bakhom, Samuel F. 2018. "Chromosomal instability drives metastasis through a cytosolic DNA response." *Nature* 553: 467-472.

Carter, Scott L., Kristian Cibulskis, Elena Helman, Aaron McKenna, Hui Shen, Travis Zack, Peter W Laird, Robert C Onofrio, Wendy Winckler, and Barbara A Weir. 2012. "Absolute quantification of somatic DNA alterations in human cancer." *Nature Biotechnology* 30: 413-421.

Chollet, François. 2017. "Xception: Deep Learning with Depthwise Separable Convolutions." *IEEE Conference on Computer Vision and Pattern Recognition (CVPR)* DOI: 10.1109/CVPR.2017.195.

Colaprico, Antonio, Tiago C Silva, Catharina Olsen, Luciano Garofano, Claudia Cava, Davide Garolini, Thais S Sabedot, Tathiane M Malta, Stefano M Pagnotta, and Isabella Castiglioni. 2015. "TCGAbiolinks: An R/Bioconductor package for integrative analysis of TCGA data." *Nucleic Acids Research* (Nucleic Acids Research) 44 (8): e71. DOI: 10.1093/nar/gkv1507.

Courtiol, Pierre, Eric W. Tramel, Marc Sanselme, and Gilles Wainrib. 2018. "Classification and disease Localization in histopathology using only global labels: A weakly-supervised approach." *arXiv: 1802.02212.*

Deng, Jia, Wei Dong, Richard Socher, Li-Jia Li, Kai Li, and Li Fei-Fei. 2009. "ImageNet: A large-scale hierarchical image database." *IEEE Conference on Computer Vision and Pattern Recognition* DOI: 10.1109/CVPR.2009.5206848.

Gamper, Jevgenij, Navid Alemi Koohbanani, Ksenija Benes, Simon Graham, Mostafa Jahanifar, Syed Ali Khurram, Ayesha Azam, Katherine Hewitt, and Nasir Rajpoot. 2020. "PanNuke Dataset Extension, Insights and Baselines." *arXiv preprint arXiv:2003.10778.*

Gamper, Jevgenij, Navid Alemi Koohbanani, Ksenija Benet, Ali Khuram, and Nasir Rajpoot. 2019. "PanNuke: an open pan-cancer histology dataset for nuclei instance segmentation and classification." In *European Congress on Digital Pathology*, 11-19. Springer.

Graham, Simon, Quoc Dang Vu, Shan E Ahmed Raza, Ayesha Azam, Yee Wah Tsang, Jin Tae Kwak, and Nasir Rajpoot. 2019. "Hover-Net: Simultaneous segmentation and classification of nuclei in multitissue histology images." *Medical Image Analysis* 58.

Gutman, David A, Mohammed Khalilia, Sanghoon Lee, Michael Nalisnik, Zach Mullen, Jonathan Beezley, Deepak R Chittajallu, David Manthey, and Lee A D Cooper. 2017. "The digital slide archive: a software platform for management, integration and analysis of histology for cancer research." *Cancer Research* 77: e75-e78; DOI: 10.1158/0008-5472.CAN-17-0629.

He, Kaiming, Xiangyu Zhang, Shaoqing Ren, and Jian Sun. 2016, DOI: 10.1109/CVPR.2016.90. "Deep Residual Learning for Image Recognition." *IEEE Conference on Computer Vision and Pattern Recognition (CVPR).*

He, Kaiming, Xiangyu Zhang, Shaoqing Ren, and Jian Sun. 2015. "Delving Deep into Rectifiers: Surpassing Human-Level Performance on ImageNet Classification." *ICCV* 1026-1034.

Hothorn, Torsten, and Berthold Lausen. 2003. "On the exact distribution of maximally selected rank statistics." *Computational Statistics & Data Analysis* 43 (2): 121-137.

Huang, Gao, Zhuang Liu, Laurens Van Der Maaten, and Kilian Q. Weinberger. 2017, DOI: 10.1109/CVPR.2017.243. "Densely Connected Convolutional Networks." *IEEE Conference on Pattern Recognition and Computer Vision (CVPR).*

Korotkevich, Gennady, Vladimir Sukhov, Alexey Sergushichev, Boris Shpak, Maxim N. Artyomov, and Alexey Sergushichev. 2019, DOI: https://doi.org/10.1101/060012. "Fast gene set enrichment analysis." *bioRxiv.*

Lausen, Berthold, Torsten Hothorn, Frank Bretz, and Martin Schumacher. 2004. "Assessment of Optimal Selected Prognostic Factors." *Biomedical Journal* 46 (3): 364-374.

Prechelt , Lutz. 2012. "Early Stopping - But When?" In *Neural Networks: Tricks of the Trade*, 53-67. Springer.

Ritchie, Matthew E., Belinda Phipson, Di Wu, Yifang Hu, Charity W. Law, Wei Shi, and Gordon K. Smyth. 2015. "Limma powers differential expression analyses for RNA-sequencing and microaary studies." *Nucleic Acids Research* 43 (7): e47, https://doi.org/10.1093/nar/gkv007.

Salas, Lucas A., Kevin C. Johnson, Devin C Koestler, Dylan E O'Sullivan, and Brock C Christensen. 2017. "Integrative epigenetic and genetic pan-cancer somatic alteration portraits." *Epigenetics* 12 (7): 561-574.

test. n.d.

Weinstein, John N, Eric A Collisson, Gordon B Mills, Kenna R Mills Shaw, Brad A Ozenberger, Kyle Ellrott, Ilya Shmulevich, Chris Sander, and Joshua M Stuart. 2013. "The cancer genome AtlansPan-Cancer analysis project." *Nature Genetics* 45: 1113-1120.
